# Application of ozone during incubation period: hatchability, chick quality and organ growth, bacterial load of feces, and first-week performance in broilers

**DOI:** 10.1007/s11259-025-10702-2

**Published:** 2025-03-08

**Authors:** Bilgehan Yılmaz Dikmen, Arda Sözcü, Aydın İpek

**Affiliations:** Department of Animal Science, Faculty of Agriculture, Bursa Uludağ University, Bursa, Türkiye

**Keywords:** Ozone, Hatchability, Hatch window, Growing performance, Broiler

## Abstract

This study was aimed to investigate the effects of ozone (O_3_) treatment during incubation period (IP) on hatchability, hatch window, chick quality and organ growth, bacterial load of feces and first-week growth performance in broilers. A total of 240 hatching eggs were weighed and randomly divided into control group (O_3_-IP (-)) and O_3_ treatment (O_3_-IP (+)). A commercial O_3_ generator was placed into the setter and O_3_ treatment (at the level of 0.050 ppm) was applied during 1 min per hour in a cyclic period of 3 days during the 18-day incubation period. The egg weight loss between 1 and 18 days ranged with values 8.59% in O_3_-IP (-) and 10.63% in O_3_-IP (+) group. The pipping time and incubation length was determined as 500.67 h and 527.33 h in O_3_-IP (-) and 489.67 h and 518.33 h in O_3_-IP (+) respectively. The yolk sac weight was found to be higher in the O_3_-IP (-) group compared to the O_3_-IP (+). In conclusion, O_3_ treatment during incubation period seems to be cause an acceleration for pipping time and shortening of total incubation period, unsteady effects for chick growth and quality, inhibitory effect for bacterial growth in feces.

## Introduction

Under modern production conditions of the poultry industry, incubation has crucial importance to obtain the high quality of one-day-old chicks. The chick quality, including health status and survivability, is affected by various factors, such as breeder genotype, age, nutrition, flock health, quality of hatching eggs, egg handling and processing procedure and incubation conditions (Bergoug et al. 2013; Wlazlo et al. 2020; Ipek and Sözcü 2015). In this respect, due to many critical factors for chick quality and performance, scientific and technological attempts recently have been carried out to develop innovative methods for egg handling, incubation modes, and biological control methods (Daraei et al. 2013; Gogaev et al. 2021).

One of the most important critical issues in hatchery management guides is sanitary conditions disinfection techniques of hatching eggs (Oliveira et al. 2022). This procedure is essential to prevent of pathogenic microbial infection during incubation period, by reducing the pathogenic microbiata on eggshells. It has been reported that the eggshell surface comprises various microorganisms, including *Escherichia coli*, *Salmonella*, *Streptococcus*, *Staphylococcus*, *Yersinia*,* Micrococcus*,* Achromobacter*,* Aerobacter*,* Alcaligenes*,* Arthrobacter*,* Bacillus*,* Cytophaga*,* Flavobacterium*,* Pseudomonas*,* Aeromonas*,* Proteus*,* Sarcina*,* and Serratia* (Mayes and Takeballi 1983; Jones et al. 2004; Musgrove et al. 2008). For the sanitation of egg surface, synthetic products such as hydrogen peroxide, formaldehyde are used in routine process in hatcheries (Gholami-Ahangaran et al. 2016). Despite these active substances are non-toxic, non-corrosive and non-damaging to the eggshells, some undesirable effects could be observed, by causing severe toxicity to developing embryo in egg and causing their death as a result of formaldehyde sanitation of eggs (Gholami-Ahangaran et al. 2016; Gogaev et al. 2021).

In routine procedure of hathing eggs sanitation, the formaldehyde is the most commonly used sanitizer in European countries, and also other countries (Gholami-Ahangaran et al. 2016; Oliveira et al. 2021, 2022). Due to high risk of hazardous formaldehyde exposure, a short exposure that is not exceeding 0.1 mg/m^3^ (0.08 ppm) of formaldehyde is recommended to protect the human health. However, the requirement of formaldehyde to reduce the microbial load of eggshell should be at least 600 mg/m^3^ (489 ppm) concentration, which is critically excessive than upper limit for human health (Cadirci 2009).

According to information mentioned above, a huge interest have recently appeared for natural products for egg sanitatiton, for example, propolis (Batkowska et al. 2018a), clove essential oils (Oliveira et al. 2020a, b), red grapefruit juice (Batkowska et al. 2018b), alicine (Çopur et al. 2011). Beside, in recent years, an innovative approach for sanitation process have been became a current issue, sanitation with ozone (O_3_) due to its strongest disinfectant effect (Gogaev et al. 2021). The O_3_ is a bluish gas with a characteristic odour (Vitali and Valdenassi 2019). It has some advantages due to its short half-life, having ability for converting into oxygen without any residue, and its strong effect for antiviral activity, antibacterial activity, destructive activity on algae, protozoa, fungi spores and cysts (Vitali and Valdenassi 2019). Therefore, it is already largely used as a disinfectant for drinking water, food industry, refrigeration water and also in wastewater treatment (Macauley et al. 2006).

O_3_ therapy has been used in human medicine for a very long time (Andres-Cano et al. 2016; Meng et al. 2017). In medicine, O_3_ can repair damaged tissue by providing sufficient energy and oxygen to injured tissues because it is ten times more soluble than oxygen, and can protect blood cells from oxidative stress by increasing the oxygen concentration in the blood (Giunta et al. 2001; Wang et al. 2014). Oxidative stress potentially causes growth retardation during post-natal and posthatch period, malformations, and embryonic death in both mammalian and avian species (Dennery 2007; Haussmann et al. 2012). There are limited studies that focus on the application of ozonization technology during incubation. According to these studies, the O_3_-applied group experienced a decrease in total microbial contamination, an improvement in embryo development, and even an increase in embryo growth (Timchenko et al. 2024); the incubation environment’s air dust concentration also decreased, and the number of healthy chicks increased (Vozmilov et al. 2019).

On the other hand, in animal production ozonization may lessen bacterial or ammonia-affected disorders, which would benefit animal producers financially, if it increases or sustains output levels, is safe for both farmers and birds, and lowers ammonia and bacterial levels. Thus, various ozonization technology is being developed and introduced to the poultry sector, focusing on ozonization in intensive animal production units to minimize odor, atmospheric ammonia levels, and bacterial load (Schwean-Lardner et al. 2009).

The aim of this study was to determine the effects of O_3_ application during incubation period on egg shell microbial load, hatchability, chick quality parameters and faeces microbial load of one-day old broiler chicks. We also evaluated the effects of O_3_ application during growing period on broiler growth performance during first week. More specifically, we hypothesize that: (1) the O_3_ application during incubation period could have an inhibiting effect for bacterial load which potentially affect embryonic mortality and hatchability, (2) the O_3_ could have an ability to stimulate embryonic development, due to its high activity, (3) the O_3_ with its strong antibacterial effect, could positively effect navel status of newly hatched chicks and the bacterial load of faeces, (4) during post-hatch period, the O_3_ treatment could affect growth performance, bacterial load of faeces.

## Materials and methods

### Ethical statement

This study was conducted at Bursa Uludağ University Faculty of Agriculture Research and Application Unit. The Animal Use and Ethical Committee of Uludağ University approved the care and use of animals for research purposes, ensuring compliance with Turkish laws and regulations (Approval Number 2023-12/02).

### Incubation period

The study employed 240 eggs from Ross 308 broiler breeder flocks that were 45 weeks old. The non-fumigated eggs (68.8 ± 0.3 g) were randomly divided into two groups: control group and O_3_ application group (n: 3 trays/application group, 40 eggs/tray). The eggs were weight to determine the egg setting weight (ESW) and then placed in two fully-automated setters (for control group and O_3_ group) with identical features, which were calibrated prior to the experiment (640 capacity egg setter, T640 I, Çimuka Inc., Ankara, Türkiye). A commercial O_3_ generator was placed inside of the setter, with O_3_ gas generated for 1 min every hour to provide O_3_ gas at a concentration of 0.050 ppm (Otrica VH-510X Pro Ozone Generator, İstanbul, Türkiye). A volume-based O_3_ application was planned, taking into account the size of the setter. Throughout the 18-day incubation phase, O_3_ gas was applied in three-day cycles. Setters were maintained at 37.2–37.5ºC temperature and 55% relative humidity during to incubation period. At the end of the 18th day all eggs were weighed with a balance with ± 0.1 g precision to determine the egg transfer weight (ETW) and calculate the egg weight loss (EWL). The trays were coded and transferred to the hatcher (640 egg capacity hatcher, T640 H, Çimuka Inc., Ankara, Türkiye). During the hatching period (18–21 days of incubation), the eggs were incubated with 36.8–37.0ºC and 70–75% relative humidity. The EWL was calculated using the following formula:

$$\:EWL\:\left(\%\right)=\left(\right(ESW\--ETW)/ESW)\times\:100$$

To determine the hatch window, the eggs in the setters were monitored at 8-hour intervals, starting from the 432nd hour of incubation, until hatching was completed. During this period, the time for first pipping was recorded for each application group. For each of observation time, the counted number of chicks was recorded to calculate the percentage of hatched chicks by initial number of eggs. All hatched chicks were weighed with a precision scale of ± 0.01 g to determine the chick hatching weight. The chicks were classified as saleable or cull chick (crippled, abnormal, belly not closed, leg problems, etc.) (Tona et al. 2004). The percentage of saleable and cull chicks was expressed as a percentage of fertile eggs (Molenaar et al. 2011). All hatched chicks were scored for navel condition according to this scoring system: score 1 - a clean and closed navel, score 2 - a black, button or gap smaller than 2 mm, or score 3 - a black button or gap larger than 2 mm (Molenaar et al. 2011). The mean value of navel score was given for each treatment group.

After completion of hatching process, the total time of incubation period for the application groups was determined and the unhatched eggs were opened to macroscopically one by one to identify fertility, contamination or embryonic mortality (early term mortality during the first week of incubation, middle term mortality between 8 and 18 d of incubation, late term mortality between 19 and 21 d of incubation) (Sözcü et al. 2022). Using the data obtained; fertility rate, embryonic mortality rate, hatchability of total eggs, hatchability of fertile eggs, were calculated (Fasenko et al. 2009). Hatchability of fertile eggs was expressed as a ratio between the number of hatched saleable chicks and the number of set fertile eggs.

On the hatching day, chick quality was determined by scoring of 15 male and 15 female chicks (30 chicks in total from each application group) randomly selected (Tona et al. 2004; Molenaar et al. 2011). Then, chick quality was determined taking into account quality criteria such as whether the belly was closed or not, liveliness and activity. The chick length was measured with a ± 0.01 mm digital caliper, from the tip of the beak to the tip of the longest toe by placing the chick face down on a flat surface and straightening the left leg (Hill 2001).

Also, randomly sampled 12 chicks from each application group used for determination of chick weight, yolk sac weight, crop, gizzard, heart and liver weight (n: 12 chicks/application group). The chicks from each treatment group were killed by cervical dislocation to obtain yolk sac, yolk-free body, crop, gizzard, heart and liver (Willemsen et al. 2010).

1 $$\begin{array}{l}Relative\:yolk\:sac\:weight\:\left(\%\right)=\\\left(yolk\:sac\:weight\:\right(g)/\:chick\:weight(g\left)\right)\times\:100\\\:Yolk\text{-}free\:Body\:Weight\:\left(g\right)=\\chick\:weight\:\left(g\right)-\:yolk\:sac\:weight\:\left(g\right)\end{array}$$

Furthermore, randomly sampled 5 chicks from each application group were put into clean hatching basket to collect the feces samples for microbiological analysis including total mesophilic aerobic bacteria (TMAB), total coliform, mold and yeast. The fecal matter were transferred into sterile sample containers after immediately defecation.

### Growing period

**Fig. 1:**
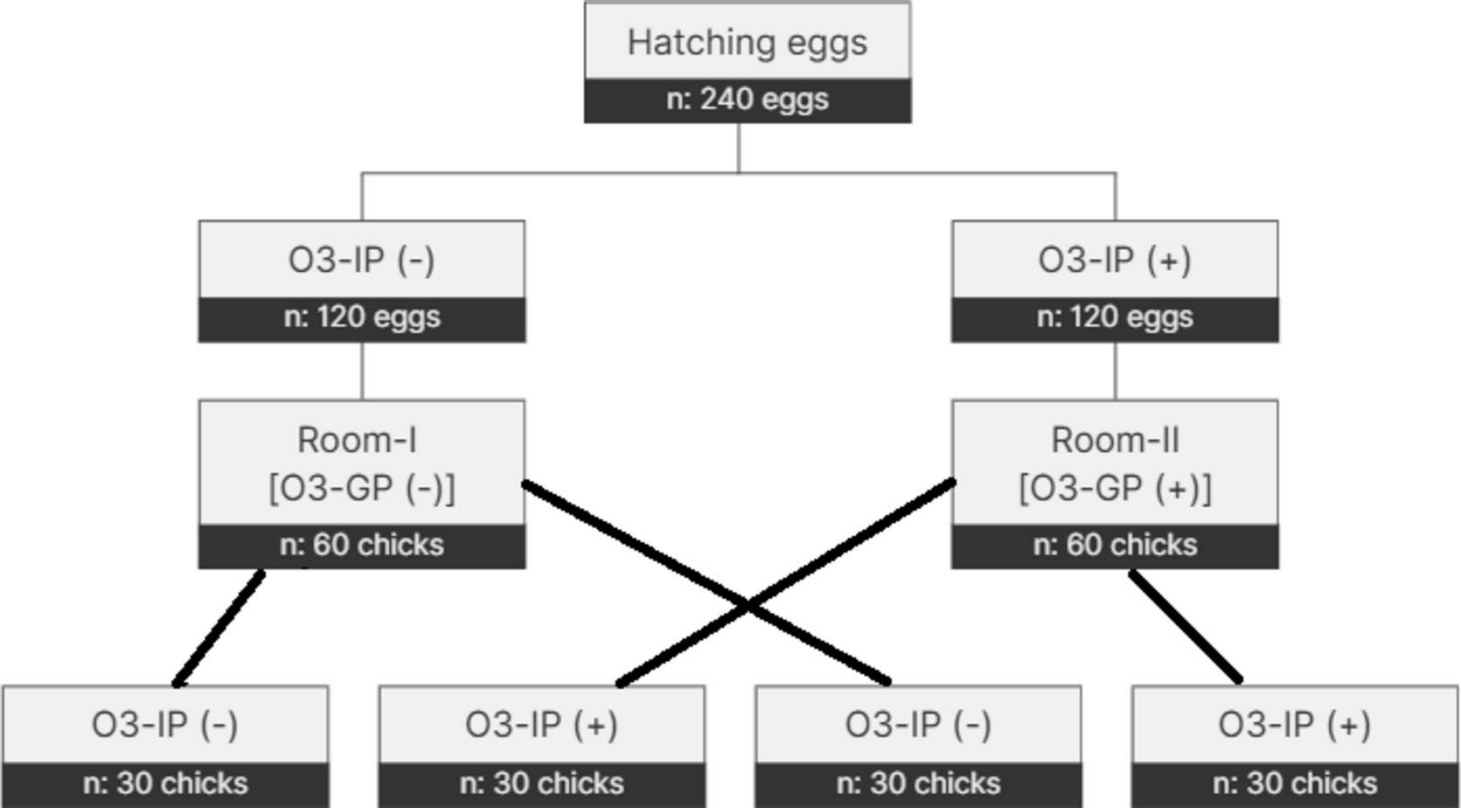
A schematic diagram of the study by O_3_ application during the incubation period (O_3_-IP) and the growing period (O_3_-GP); O_3_-IP (-): control group during incubation period; O_3_-IP (+): Ozone treatment group during incubation period

A total of 120 chicks were divided into 4 groups (as shown in Fig. 1), according to the O_3_ application during incubation and growth period, with three replications in each treatment group, consisting of 10 chicks per pen, were placed (n: 10 chicks/pen, 3 pens/treatment group). In the study, two growth rooms of equal conditions and size were used as control and O_3_ application. In one of these rooms, the O_3_ generator was activated for 10 min every 8 h for 7 days and applied 0.050 ppm O_3_ into the room (Otrica VH-510X Pro Ozone Generator, İstanbul, Türkiye).

The day-old chicks were feather sexed, and then weighed using a balance at ± 0.1 g precision on the first day of the growing period. Wood shavings laid at a thickness of 8 to 10 cm on the floors of the pens were used as litter material.

The chicks received a standard pelleted broiler starter diet (22.5% CP and ME 12.8 MJ/kg of diet) between days 1 to 7 days. During experimental period, feed and water were offered ad libitum. The chicks were exposed to 23 h of light and 1 h of darkness (30 to 40 lx/m^2^). Room temperature was 33 ⁰C at 1 d of age during the first days, and then gradually decreased to 28–30 ⁰C until the end of the 1st week. The CO_2_ concentrations were monitored using an INNOVA 1314i photoacoustic multi-gas monitor 1314i (LumaSense Technologies A/S, Ballerup, Denmark). The data was given as daily average value of CO_2_ concentration for O_3_-GP (+) and O_3_-GP (-) rooms.

At the end of the first week, the chicks were weighed and their feed consumption was determined on a group basis, live weight gain and feed conversion ratio (FCR) were calculated. The FCR was calculated on pen basis using the weekly live weight gains and feed consumption values. Mortality by pen was recorded daily.

At the end of the one-week growing period, randomly selected 3 chicks from each treatment group were randomly taken into clean pens. The chicks’ faeces were taken into sterile sample containers after immediately defecation, for microbial analysis including total mesophilic aerobic bacteria, total coliform, mold and yeast.

### Microbial analysis

The faeces samples with an amount of 10 g were placed in sterile containers containing 50 mL of phosphate buffered saline solution, and homogenized for 2 min with a vortex. To numerate of microorganisms in samples, the decimal dilutions were prepared in the tubes with 9 ml of 0.1% phosphate buffered saline.

For enumeration of TMAB and coliforms, Plate Count Agar (PCA) and Violet Red Bile Agar (VRB, Merck, Germany) were used respectively. Duplicate pour plates were made from each dilution. Plates were incubated at 35˚C during 48 h for PCA, at 30˚C during 24 h for VRB. All colonies from the appropriate dilution were counted as mesophilic aerobic bacteria, and pink-red colonies from the appropriate dilution were counted as coliform bacteria (Harrigan 1998).

To determine the count of mold and yeast, 10% tartaric acid added Potato Dextrose Agar (PDA) was used. Duplicate pour plates were made from each dilution, and then the plates were incubated for five days at 22 ˚C (Andrew 1992). Following the incubation, the colonies with soft mucoid consistency, oval or rounded edges were evaluated as yeast, whereas those with a “puffy cotton” mycelium appearance were evaluated as mold.

### Statistical analysis

The study was conducted on a 2 × 2 factorial trial design and data was analysed by analysis of variance using General Linear Models (Minitab 2013). Analysis of percentage data were conducted after arcsine square root transformation of the data. For incubation period data differences in investigated traits were analysed by two samples T-test (Minitab 2013). For the growing period data differences in investigated traits according to the O_3_ application during the incubation period and the growing period and their interactions were analysed by Two-way ANOVA (Minitab 2013). Data were presented as mean ± standard error in all of the tables and figures. Differences were considered significant at *P* ≤ 0.05 and the statistical difference at *P* < 0.10 was described as a tendency.

## Results

The effect of O_3_ application on incubation results are presented in Table 1. The EWL was found to be higher in O_3_-IP (+) group than the O_3_-IP (-) (10.63% vs. 8.59%, *P* = 0.006). The fertility, hatchability of total and fertile eggs, early term, midterm, late term embryonic mortality and pip mortality were found to be similar between the O_3_-IP (-) and O_3_-IP (+) groups (*P* > 0.05). Similar mean values for the percentage of contaminated egg and cull chicks, chick hatching weight and chick yield were observed for both the O_3_-IP (-) and O_3_-IP (+) groups (*P* > 0.05). On the other hand, significant differences were found for pipping time and total incubation length in O_3_-IP (-) (500.67 h and 527.33 h respectively) and O_3_-IP (+) (489.67 h and 518.33 h respectively) groups (*P* < 0.05).

**Table 1:** The effect of O_3_ application during incubation period on incubation results

Incubation traits	O_3_-IP (-)	O_3_-IP (+)	*P* - *Value*
EWL, %	8.59 ± 0.09^b^	10.63 ± 0.26^a^	*0.006*
Fertility, %	82.85 ± 7.93	81.92 ± 8.47	*NS*
Hatchability of total eggs, %	64.57 ± 4.34	59.99 ± 4.74	*NS*
Hatchability of fertile eggs, %	78.13 ± 4.01	74.40 ± 3.32	*NS*
Early Embryo Mortality, %	9.51 ± 7.71	12.7 ± 10.5	*NS*
Mid Embryo Mortality, %	3.45	**-**	*-*
Late Embryo Mortality, %	9.06 ± 5.50	10.88 ± 4.03	*NS*
Pip Mortality, %	1.15 ± 1.99	1.01 ± 1.75	*NS*
Contaminated egg, %	2.16 ± 1.88	1.01 ± 1.75	*NS*
Cull chick,%	1.01 ± 1.75	1.01 ± 1.75	*NS*
Chick hatching weight, g	48.39 ± 1.08	46.97 ± 0.91	*NS*
Chick yield,%	70.28 ± 1.39	68.30 ± 1.08	*NS*
Pipping time (h)	500.67 ± 3.06^a^	489.67 ± 4.51^b^	*0.025*
Incubation length (h)	527.33 ± 2.08^a^	518.33 ± 3.51^b^	*0.019*

*NS* Not significant

^a, b^ Values with different superscripts in the same column differ statistically (*P* < 0.05)

**Fig. 2:**
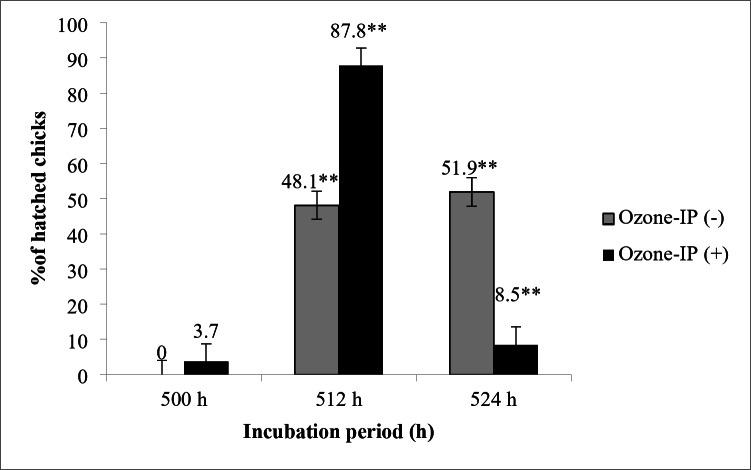
The effect of O_3_ application during incubation period on hatch window. Bars represent mean ± SE (** *P* < 0.01)

The effect of O_3_ application during incubation period on hatch window results is presented in Fig. 2. At the 500 h of incubation 3.7% chicks hatched in O_3_-IP (+) group and any chicks were hatched in the O_3_-IP (-) group (*P* > 0.05). At the 512 h of incubation, the percentage of hatched chicks was found significantly higher in O_3_-IP (+) group than O_3_-IP (-) group (87.8% vs. 48.1%; *P* = 0.003), whereas it was found to be higher in the O_3_-IP (-) group than O_3_-IP (+) group at the 524 h of incubation (8.5% vs. 52.1%; *P* = 0.008).

The effect of O_3_ application during incubation on chick quality parameters at hatch are presented in Table 2. The chick weight and yolk sac weight were found to be significantly higher in O_3_-IP (-) with values of 47.99 g and 5.37 g respectively, than the O_3_-IP (+) group (45.89 g and 3.58 g, *P* < 0.05). On the other hand, a higher mean value for relative weight of crop and gizzard were observed in O_3_-IP (+) group (0.86% and 6.10%, *P* < 0.05), whereas the relative weight of heart was found to be higher in the O_3_-IP (-) group compared to the O_3_-IP (+) (0.95% vs. 0.78%, *P* < 0.05).

**Table 2:** The effect of O_3_ application during incubation on chick quality parameters at hatch

Chick Parameters	O_3_-IP (-)	O_3_-IP (+)	*P* - *Value*
Chick weight, g	47.99 ± 0.87^a^	45.89 ± 0.79^b^	*0.002*
Chick length, cm	20.08 ± 0.67	20.72 ± 0.31	*0.081*
Navel score	1.41 ± 0.59	1.36 ± 0.66	*NS*
Yolk sac weight (g)	5.37 ± 1.57^a^	3.58 ± 1.15^b^	*0.050*
Relative yolk sac weight (%)	11.20 ± 3.30	7.81 ± 2.55	*0.077*
Yolk-free body weight (g)	42.62 ± 1.92	42.31 ± 1.52	*ns*
Crop (%)	0.74 ± 0.05^b^	0.86 ± 0.09^a^	*0.030*
Gizzard (%)	4.78 ± 0.40^b^	6.10 ± 0.83^a^	*0.010*
Heart (%)	0.95 ± 0.11^a^	0.78 ± 0.09^b^	*0.016*
Liver (%)	2.82 ± 0.15	3.14 ± 0.34	*0.077*

*NS* Not significant

^a, b^ Values with different superscripts in the same column differ statistically (*P* < 0.05)

**Fig. 3:**
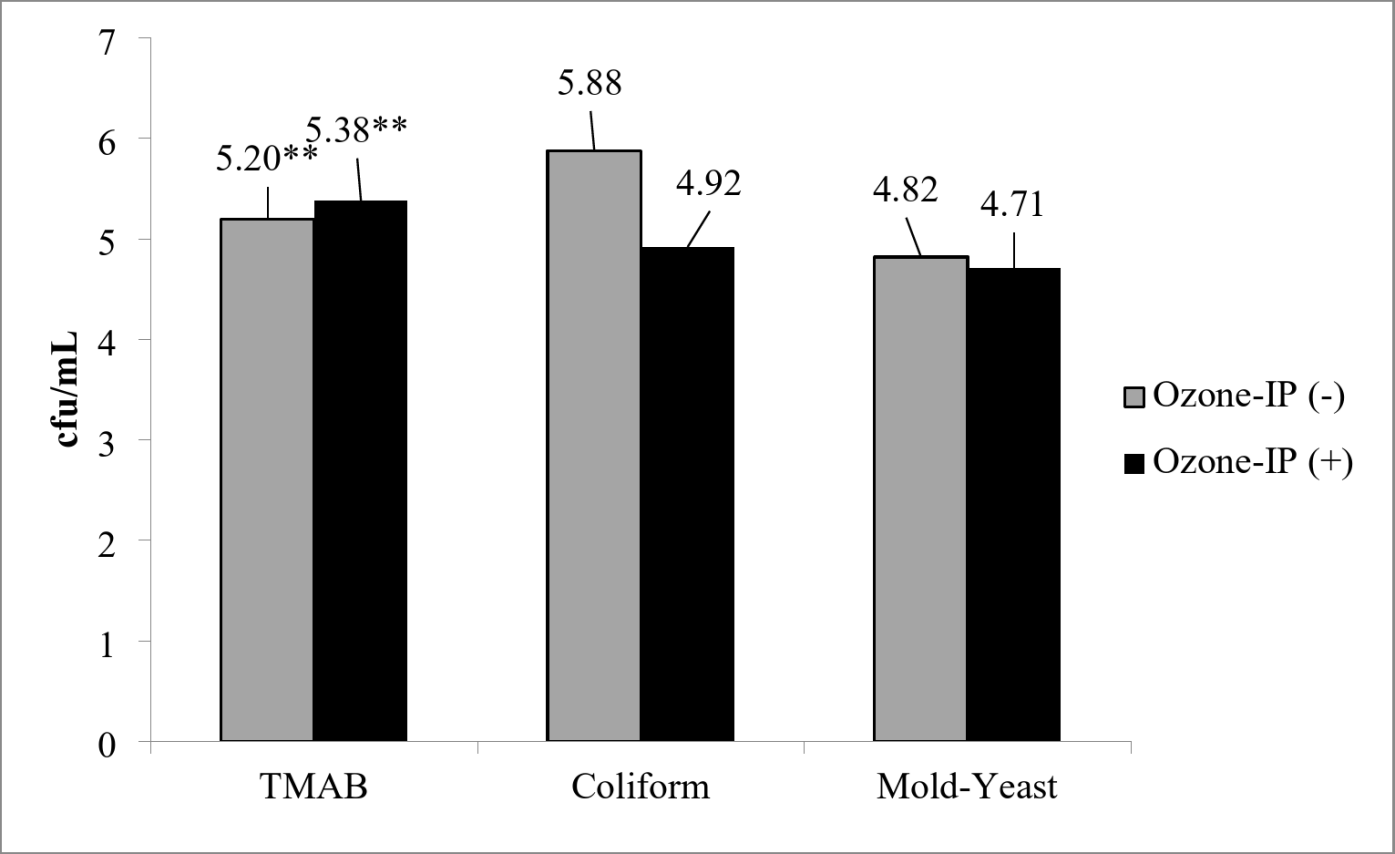
The effect of O_3_ application during incubation period on faeces microbial load at hatch. Bars represent mean ± SE (** *P* < 0.01)

The effect of O_3_ application on faeces microbial load at hatch is presented in Fig. 3. The number of TMAB was found to be higher in O_3_-IP (+) than O_3_-IP (-) group (5.38 *versus* 5.20 respectively; *P* < 0.01). The number of *Coliform sp*. and yeast mold count at hatch were found similar between the groups (*P* > 0.05).

The effect of O_3_ application on the first-week growth performance of broilers is presented in Table 3. No any significant effects of incubation × growing period of O_3_ treatments were observed for growth performance (*P* > 0.05). The effect of O_3_-IP (+) on body weight of chicks at day 1 and day 7, body weight gain were found to be higher O_3_-IP (-) group (*P* < 0.05). The feed consumption and FCR were found to be similar between the groups (*P* > 0.05). On the other hand, O_3_-GP (+) caused no any significant effects for growth performance of broilers. Furhermore, there was no mortality in the trial groups during the first week of growing period.

**Table 3:** The effect of O_3_ application on first week growth performance of broilers

O_3_ treatments	Body weight		Body weight gain	Feed consumption	FCR
	Day 1	Day 7			
Incubation treatment					
O_3_-IP (-)	47.51^a^	182.13^a^	134.63^a^	165.97	0.91
O_3_-IP (+)	46.68^b^	173.78^b^	127.11^b^	159.95	0.92
SEM	0.21	1.49	1.48	5.29	0.03
*P-Value*	*0.048*	*0.017*	*0.023*	*NS*	*NS*
Growing period treatment					
O_3_-GP (-)	47.57^a^	175.98	128.42	166.82	0.95
O_3_-GP (+)	46.62^b^	179.93	133.32	159.10	0.88
SEM	0.21	1.49	1.48	5.29	0.03
*P-Value*	*0.031*	*NS*	*NS*	*NS*	*NS*
Incubation × Growing period					
O_3_ IP (-) ***×*** GP (-)	47.94	179.54	131.60	169.27	0.94
O_3_ IP (-) ***×*** GP (+)	47.08	184.73	137.66	162.67	0.88
O_3_ IP (+) ***×*** GP (-)	47.20	172.43	125.24	164.37	0.95
O_3_ IP (+) ***×*** GP (+)	46.18	175.13	128.98	155.53	0.89
SEM	0.29	2.10	2.10	7.48	0.04
*P-Value*	*NS*	*NS*	*NS*	*NS*	*NS*

*NS* Not significant

^a, b^ Values with different superscripts in the same column differ statistically (*P* < 0.05)

The effect of O_3_ application on faeces’ microbial load of broilers at 7 days of age is presented in Table 4. No any significant effects of O_3_ application during incubation period was observed on the count of TMAB of faeces at 7 days of age (*P* > 0.05). However; the counts of *Coliform sp*. and yeast- mold count were found to be higher in O_3_-IP (-) than in O_3_-IP (+) group (*P* < 0.001).

**Table 4:** The effect of O_3_ application on faeces microbial load of broilers at 7 days of age

O_3_ treatments	TMAB (cfu/mL)	*Coliform sp.* (cfu/mL)	Mold-yeast (cfu/mL)
Incubation treatment			
O_3_-IP (-)	5.24	5.59^a^	4.89
O_3_-IP (+)	5.25	5.12^b^	4.85
SEM	0.17	0.11	0.07
*P-Value*	*NS*	*< 0.0001*	*NS*
Growing period treatment			
O_3_-GP (-)	5.08	5.49^a^	4.96^a^
O_3_-GP (+)	5.42	5.22^b^	4.78^b^
SEM	0.17	0.11	0.07
*P-Value*	*NS*	*0.025*	*0.033*
Incubation × Growing period			
O_3_ IP (-) ***×*** GP (-)	5.06	5.62	5.01
O_3_ IP (-) ***×*** GP (+)	5.42	5.57	4.77
O_3_ IP (+) ***×*** GP (-)	5.09	5.36	4.91
O_3_ IP (+) ***×*** GP (+)	5.41	4.87	4.80
SEM	0.24	0.16	0.11
*P-Value*	*NS*	*NS*	*NS*

*NS* Not significant

^a, b^ Values with different superscripts in the same column differ statistically (*P* < 0.05)

**Fig. 4:**
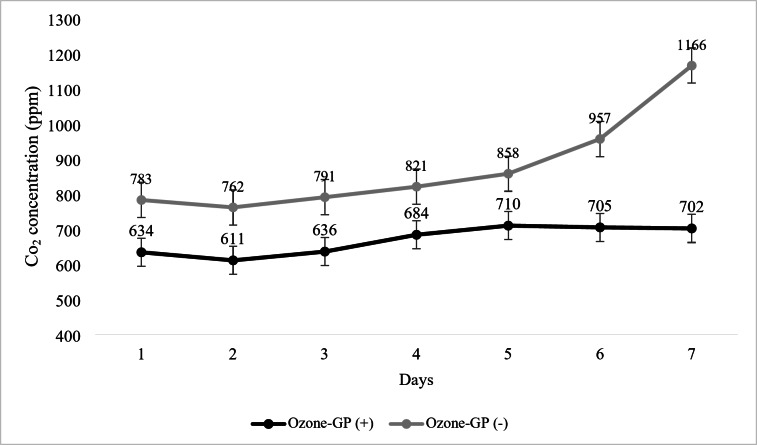
Changes in CO_2_ concentration of growth rooms by O_3_ application during growing period

Changes in CO_2_ concentration of growth rooms by O_3_ application during growing period is shown in Fig. 4. As shown in the figure, O_3_ application provided a decline in CO_2_ concentration in the room when compared to the O_3_-GP (-) room. The daily mean of CO_2_ concentration in the room changed between 611 and 710 ppm in O_3_-GP (+) and 762 and 1166 ppm O_3_-GP (-) group (*P* < 0.01).

## Discussion

It is well known that the EWL which ranges between 9.0 and 12.0% during the first cycle of the incubation period, is an important issue for hatchability, embryo development and chick quality (Romao et al. 2008; Nowaczewski et al. 2012). In the current study, the percentage of EWL during incubation ranged with values 8.59% in O_3_-IP (-) and 10.63% in O_3_-IP (+) group. This increment in EWL of O_3_-IP (+) group could be related the potential detrimental effects of O_3_ on cuticle layer of eggshell, which consequently causes, deteriorate eggshell permeability, previously suggested by Brake and Sheldon (1990). If the cuticle is damaged, the water loss by eggshell pores shows an increment (Peebles et al. 1998). In a previous study performed by Fuhrmann et al. (2010), it was stated that the treatment of low O_3_ doses with an amount of 10 ppm caused a complete destroy for cuticle proteins. On the other hand, Koç and Aygün (2021) found no any significant differences for EWL between control and O_3_ application (1 ppm, 3 ppm, 5 ppm, 7 ppm O_3_) groups. The differences observed between studies could be related to the method, duration and amount of O_3_ application.

The current study clearly showed that O_3_ treatment of hatching eggs caused no significant effects on hatchability, embryo mortality, chick hatching weight and chick yield. These results are similar with other findings reported by Melo et al. (2019); applied O_3_ 5–15 ppm/30 min fumigation before the incubation, Hrnčár et al. (2012); applied 0.450 ppm O_3_ during 12 h before the incubation, Koç and Aygün (2021); applied 1%, 3%, 5% and 7% O_3_ with generator before the incubation whom reported any effects of O_3_ treatment of hatching eggs on hatchability. Hrnčár et al. (2012) found no differences in hatchability between the eggs before the incubation disinfected with O_3_ (0.450 ppm during 12 h) and the traditional method with formalin gas (20 g KMnO_4_ + 30 g formaldehyde of 40% concentration to 1 m^3^ of an area) in Oravka chickens. Contrarily to the current and previous results, Wlazlo et al. (2020) who applied different disinfection methods (formaldehyde fumigation, perhydrol (H_2_O_2_) by spraying and ozone (4.2 mg O_3_/h, 5 min) to eggs before the incubation found that a significant decline in hatchability of fertile eggs when applied O_3_ disinfection method (64.32%) compared to the control method (80.91%) in Japanese quails. This could be accompanied with the modifying effect of O_3_ on egg composition, in the way of reducing of vitamin A and vitamin E content, and fatty acid profile of egg yolk, which subsequently cause an inhibition for embryo development (Fuhrmann et al. 2010). The results of embryo mortality, chick hatching weight and chick yield of eggs treated with O_3_ during the incubation period did not differ from those obtained in the control groups. However, Wlazlo et al. (2020) who applied O_3_ disinfection method to eggs before the incubation found a significant increment in embryo mortality due to negative effect of O_3_ treatment. On the other hand, Vozmilov et al. (2019) used an electro-filtration system with increased O_3_ generation based on corona discharge for continuous disinfection of both the air and the surface of the eggs in the setter by ozonization technology and O_3_ concentration during incubation ranged between 5.58 and 7.7 mg/m^3^ reported that percentage of healthy chicks in the O_3_-applied group was 3.44% greater than that of the control setter. The way, duration and concentration of O_3_ application in the studies may affect the incubation parameters, which may explain the difference between our findings and the findings in the literature.

An interesting result related with pipping time and incubation length was observed in the current study, as an accelerating effect of O_3_ for hatching process of chicks when compared to the control group. It is well known that metabolism of poultry is related with the intensity of transport functions of circulating blood (Gogaev et al. 2019). Therefore, the normal process of metabolic pathways is linked to morphological and biochemical composition of bird’s blood. As a result of the exposure to O_3_, embryos could have capacity to absorb oxygen more than 40%, therefore haematopoiesis and erythropoiesis show an increment. According to these metabolic changes, embryos in O_3_ treatment group had acceleration in yolk absorption, corresponding with a lower relative yolk sac weight, although no significant differences among the treatment groups. As a result, O_3_ treatment caused a forced early hatching of chicks compared to the control chicks. As seen in the Figs. 2 and 87.8% of chicks in O_3_ treatment group hatched until 512 h of incubation period, whereas 48.1% of chicks hatched in the control group. These findings clearly showed that O_3_ also affected the hatch window range in broiler chicks. Thus, Timchenko et al. (2024) who applied O_3_ to Hysex Brown eggs with a portable ozoniser at a concentration of 2.0 mg/l for 30 min (before incubation, on the 3rd and 5th day of incubation) showed that O_3_ application did not adversely affect embryo development and even stimulated embryo growth according to micro tomographic and histological evaluations.

On the other hand, development of organs seems to be affected by O_3_ treatment during incubation. Although there was no significant for yolk-free body weight and relative yolk sac weight between control and O_3_ treatment groups, the percentage of crop, gizzard and heart weight at hatch were comparable between the treatments. The results found as higher percentage of crop and gizzard weight in O_3_ treatment, may suggest that functionality of intestinal tract of chicks was stimulated by more yolk utilisation (a smaller yolk sac weight in amount and percentage). However, functionality of heart should be impaired by O_3_ treatment during incubation period, which might cause serious health problems during post-hatch period, such as ascites. These findings suggest that O_3_ exposure of embryos during developmental stage caused a non-uniform effect on different organs.

O_3_ treatment during incubation period could potentially cause a hyperoxia for developing embryos, which have resulted in organ growth of one-day old chicks at hatch. These differences could be potentially attributed to the changes in blood flow of organs, organ specific changes in oxygen consumption, releasing of adenosine triphosphate and secretion of insulin-like growth factors, caused by hyperoxia (Asson-Batres et al. 1989; Van Golde et al. 1998).

It is well known that the gastrointestinal microbiota has crucial role for health status and production performance of commercial birds (Fathima et al. 2022). The early colonization of gut by various bacteria’s has stimulating effect for morphological and physiological development of the gut and, susceptibility against infections. During the first week of post-hatch period, infectious triggered by pathogenic bacteria’s causes significant economic losses with a poor weight gain, worsening feed efficiency and a high mortality rate in broiler production (Yassin et al. 2009; Kemmett et al. 2014). Therefore, defining the microbial diversity and composition in faeces could be accepted a tool for spot check the gut health of broilers (Swelum et al. 2021).

When evaluating the effectiveness of O_3_ application during incubation period, attention should be given to the significant change the population of total aerobic bacteria in faeces at hatch, whereas any significant effects were observed for *Coliform spp.*, and mold and yeast population. The decline in the load of total aerobic bacteria in faeces could be related the decreasing effect of O_3_ on eggshell microbial count, which previously reported by Wlazlo et al. (2020) and Koç and Aygün (2021). Also, Timchenko et al. (2024) who applied O_3_ to Hysex Brown eggs with a portable ozonizer at a concentration of 2.0 mg/l for 30 min (before incubation and on the 3rd day of incubation) and (before incubation, on the 3rd and 5th day of incubation) showed that O_3_ application had a bacteriostatic effect by reducing the total microbial contamination level by 30% and 40%, respectively. Contrarily to these findings, Melo et al. (2019) found any significant effect O_3_ disinfection on enumeration of total aerobic bacteria eggshell surface. In the hypothesis of the study, it was expected a lower population of bacteria’s, mold and yeast in faeces of newly hatched chick, due to antimicrobial effect of O_3_. However, the current results clearly indicated that O_3_ treatment during the first 18 days of incubation could not provide a satisfactory reduction in microbial count. This could be related with increasing effects of O_3_ disinfection by a high relative humidity, as previously emphasized by Braun et al. (2011).

Addition of O_3_ during incubation period caused a significant difference for body weight and body weight gain of broilers, whereas ozonisation during growing period had no effects for the broiler growth and feed efficiency. The higher body weight observed in the control group at 7 days of age could be attributed to the initial body weight of chicks in the O_3_-IP (-) and O_3_-IP (+) groups. In a previous study performed by Schwean-Lardner et al. (2009) who added the O_3_ (on average level of 0.03 ppm during 40 days) into an intensive production unit of broilers, a significant decline in body weight gain and feed consumption, and a significant improvement in feed efficiency between 1 and 40 days were found, and subsequently it was highlighted that the usage of O_3_ could be an unacceptable procedure in commercial broiler production, due to higher incidence of morbidity and mortality, serious health problems in O_3_ treated group.

It is well known that O_3_ especially of higher levels is an effective biocide (Masaoka et al. 1982). According to Dyas et al. (1983), the O_3_ at a level of 0.3–0.9 ppm could effectively kill many kinds of bacteria species. However, there are some contrast expressions in the literature about the effective dose of O_3_ for usage with bactericidal purposes (Schwean-Lardner et al. 2009). To provide such effect, it has been suggested to apply the O_3_ higher than 1 ppm (Dyas et al. 1983; Hamelin and Chung 1974). Though, the current results clearly indicated that O_3_ treatment both incubation period and growing period tended to reduce of the number of *Coliform spp.* and mold-yeast in faeces of chicks at 7 days of age.

The harmful gases inside of the poultry houses directly affect the health status both of birds and staff, especially higher concentration of some gases, such as NH_3_ and CO_2_, cause deterioration in performance, respiratory diseases, subsequently increase production cost (Al-Kerwi et al. 2022). Due to critical importance, some studies have recently focused on the ozonisation for remediation of air quality (Kim-Yang et al. 2005; Schwean-Lardner et al. 2009; Wang et al. 2010). The available reports have proven contradictory results about the effectiveness of O_3_ for air quality. Vozmilov et al. (2019) used an electro-filtration system with increased O_3_ generation based on corona discharge for continuous disinfection of both the air and the surface of the eggs in the setter, reported that O_3_ concentration during incubation ranged between 5.58 and 7.7 mg/m^3^, the air dust concentration was dropped and the microbe concentration was dropped while the system was in operation. On the other hand, in another study, it was reported that O_3_ treatment with a concentration of 0.1 ppm caused any significant remediation of air quality, with regard to dust mass concentration, odour concentrations, sulphur compound concentrations, and bacteria population in a swine barn (Elenbass-Thomas et al. 2005) and NH_3_ concentration in commercial broiler houses (Wang et al. 2010. According to Dai et al. (2013), CO_2_ concentrations below 12,000 ppm have no significant effect on broiler performance, however concentrations over this threshold can cause reduced daily weight gain and an increase in feed conversion ratio, indicating a decline in health and productivity. Thus, current results could suggest that O_3_ treatment could potentially have mitigator effect for CO_2_ concentration of inside.

To out knowledge, there is no another study focused on the effects of O_3_ treatment during incubation period on chick quality and first week performance of broilers. As a result of this study, O_3_ treatment (at a level of 0.050 ppm O_3_, 1 min per hour as daily basis) caused a higher egg weight loss, an accelaration for pipping time and shortening of total incubation period, a lower chick weight and yolk sac weight, stimulating effect for crop and gizzard, inhibitory effect for heart development in broiler chicks. Despite the unsteady findings, the O_3_ treatment resulted in significant reductions in bacteria population which could be accepted as an inhibitory effect for bacterial growth, and also CO_2_ concentration in experimental unit, that could potentially have remediation effect for air quality. When using O_3_ treatments in hatcheries and poultry houses, one should be careful about the concentrations of O_3_ gaseous due to toxic effects. In conclusion, to use the ozonisation of hatching eggs, safe method and application schedules should be developed without negatively effecting embryo development and hatchability. Therefore, more studies should be needed to investigate the possible effects of O_3_ in a large scale regarding with commercial conditions.

## Data Availability

No datasets were generated or analysed during the current study.
